# Estimation of the depth of origin of fluids using noble gases in the surface sediments of submarine mud volcanoes off Tanegashima Island

**DOI:** 10.1038/s41598-023-31582-z

**Published:** 2023-04-06

**Authors:** Yuki Mitsutome, Tomohiro Toki, Takanori Kagoshima, Yuji Sano, Yama Tomonaga, Akira Ijiri

**Affiliations:** 1 Chemistry Biology and Marine Science, Graduate School of Science and Engineering, University of the Ryukyus, 1 Senbaru, Nishihara, Okinawa 903-0213 Japan; 2 Department of Chemistry, Biology and Marine Science, Faculty of Science, University of the Ryukyus, 1 Senbaru, Nishihara, Okinawa 903-0213 Japan; 3 Research Institute for Humanity and Nature, 457-4 Motoyama, Kamigamo, Kita-ku, Kyoto, 603-8047 Japan; 4 Graduate School of Science and Engineering, University of Toyama, 3190 Gofuku, Toyama, 930-8555 Japan; 5 Atmosphere and Ocean Research Institute, The University of Tokyo, 5-1-5 Kashiwanoha, Kashiwa, Chiba 277-8564 Japan; 6 Center for Advanced Marie Core Research, Kochi University, 200B Monobe, Nankoku, Kochi, 783-8502 Japan; 7 Eawag, Swiss Federal Institute of Aquatic Science and Technology, Überlandstrasse 133, 8600 Dübendorf, Switzerland; 8 Hydrogeology, Department of Environmental Sciences, University of Basel, Bernoullistrasse 32, 4056 Basel, Switzerland; 9 Graduate School of Maritime Sciences, Kobe University, 5-1-1 Fukaemiinamimachi, Higashinada-ku, Kobe, 658-0022 Japan; 10 Kochi Institute for Core Sample Research, Japan Agency for Marine-Earth Science and Technology (JAMSTEC), 200B Monobe, Nankoku, Kochi, 783-8502 Japan

**Keywords:** Marine chemistry, Geochemistry

## Abstract

The helium isotope ratio (^3^He/^4^He), concentration ratio of neon-20 to helium-4 (^20^Ne/^4^He), argon (Ar), krypton (Kr), and xenon (Xe) concentrations were measured in the porewater of surface sediments of several submarine mud volcanoes. From the ^3^He/^4^He values (0.18–0.93R_A_), the estimated He origin is almost 90% crustal He, with little contribution from mantle-derived He. The determined Ar, Kr, and Xe concentrations lie within the solubility equilibrium range expected for temperatures from 83 °C up to 230 °C and are consistent with the temperature range of the dehydration origin of clay minerals. Considering the geothermal gradient in the investigated region (25 °C/km), these gases are considered to have reached dissolution equilibrium at a depth of about 3.3 km to 9.2 km below the seafloor. As the depth of the plate boundary is 18 km below the seafloor, the noble gas signatures are likely to originate from the crust, not from the plate boundary. This is consistent with the results presented by the He isotope ratios.

## Introduction

Mud volcanoes carry mud, gas, water, and other dissolved substances from deep beneath the seafloor to the seafloor and then vent them out into the seawater from the seafloor surface, thus playing an important role in the material cycle at the Earth's surface^1,2^. Therefore, the study of the origin and chemical composition of water and gas in the seafloor surface layer of mud volcanoes is extremely important for a quantitative understanding of such cycles. Several studies have been conducted to estimate the depth of origin of mud and fluids in the surface layers of mud volcanoes by examining the concentration and isotopic ratio of some elements or compounds. For example, the carbon and hydrogen isotope ratios of methane in the surface sediments of mud volcanoes have been used to infer that the origin of methane is deeper than 1 to 2 km beneath the seafloor by using the geothermal gradient beneath the seafloor^3–7^. Several studies have also examined the isotopic ratios of water in the porewater of the seafloor surface layer and inferred that water that formed under temperature conditions ranging from 60 to 160 °C has migrated from 1 to 3 km below the seafloor, based on the possible dehydration origin of clay minerals^8–11^. Other studies have used geothermometers to estimate the equilibrium temperature of sediments and porewater from combinations of dissolved cation concentrations in surface porewater on the seafloor^8,12–14^. For example, Aloisi et al.^12^ estimated the maximum temperature experienced by fluids with a geothermometer using concentrations of magnesium and lithium of approximately 100 °C. Xu et al.^14^ similarly estimated the fluid's temperature of origin from the concentrations of magnesium and lithium in mud volcano surface sediments and found it to be consistent with the temperature at which the dehydration reaction of clay minerals is believed to occur. In addition, lithium and boron isotope ratios in porewater in the surface layer of mud volcanoes have been used to estimate the equilibrium temperature with the sediment^15–17^.

Helium (He) has two stable isotopes, ^3^He and ^4^He. ^3^He is thought to be a primordial component of the Earth's deep mantle, taken from the solar nebula when the Earth formed 4.6 billion years ago^18^. The He isotope ratio (^3^He/^4^He) in the upper mantle is estimated to be approximately eight times that of the atmosphere (^3^He/^4^He = 1.39 × 10^–6^)^19^. In notation, the He isotope ratio in the atmosphere is 1 R_A_, and the He isotope ratio in the mantle is 8 R_A_. On the other hand, ^4^He is produced by radioactive decay of uranium (U) and thorium (Th), the U and Th concentrations are much higher in the Earth's crust than in the mantle, and the ^3^He/^4^He ratio of crustal He is considered to be 0.02 R_A_^19^. Measurements of He isotope ratios in mud volcanoes in the Gulf of Cadiz suggest that helium (He) was mainly produced in the crust and that the emitted fluids did not originate from the plate boundary^20^. Similarly, the origin of He in seawater immediately above mud volcanoes in the Ionian Sea in southern Italy is crustal, again suggesting the rise of deep subsurface fluids that are not connected to the mantle^21^. Thus, there are still few examples of studies on noble gases in submarine mud volcanoes.

Several mud volcanoes have been found on the seafloor off Tanegashima Island^22^, where the water is thought to be from the dehydration origin of clay minerals^23^. However, no noble gas data exists on many of the mud volcanoes in the area. In this study, we estimate the contribution of mantle-derived He from the measured ^3^He/^4^He ratios in surface sediments of submarine mud volcanoes off Tanegashima Island, and discuss whether the He source is connected to the plate boundary as a possible supply route for mantle-derived He. We also identify atmospheric-derived gases using the determined ^20^Ne/^4^He ratios, and discuss the current activity status of each mud volcano. Furthermore, the concentrations of heavier noble gases (i.e., Ar, Kr, and Xe) are investigated to assess gas-partitioning processes at depth.

## Geological setting

**Figure 1:**
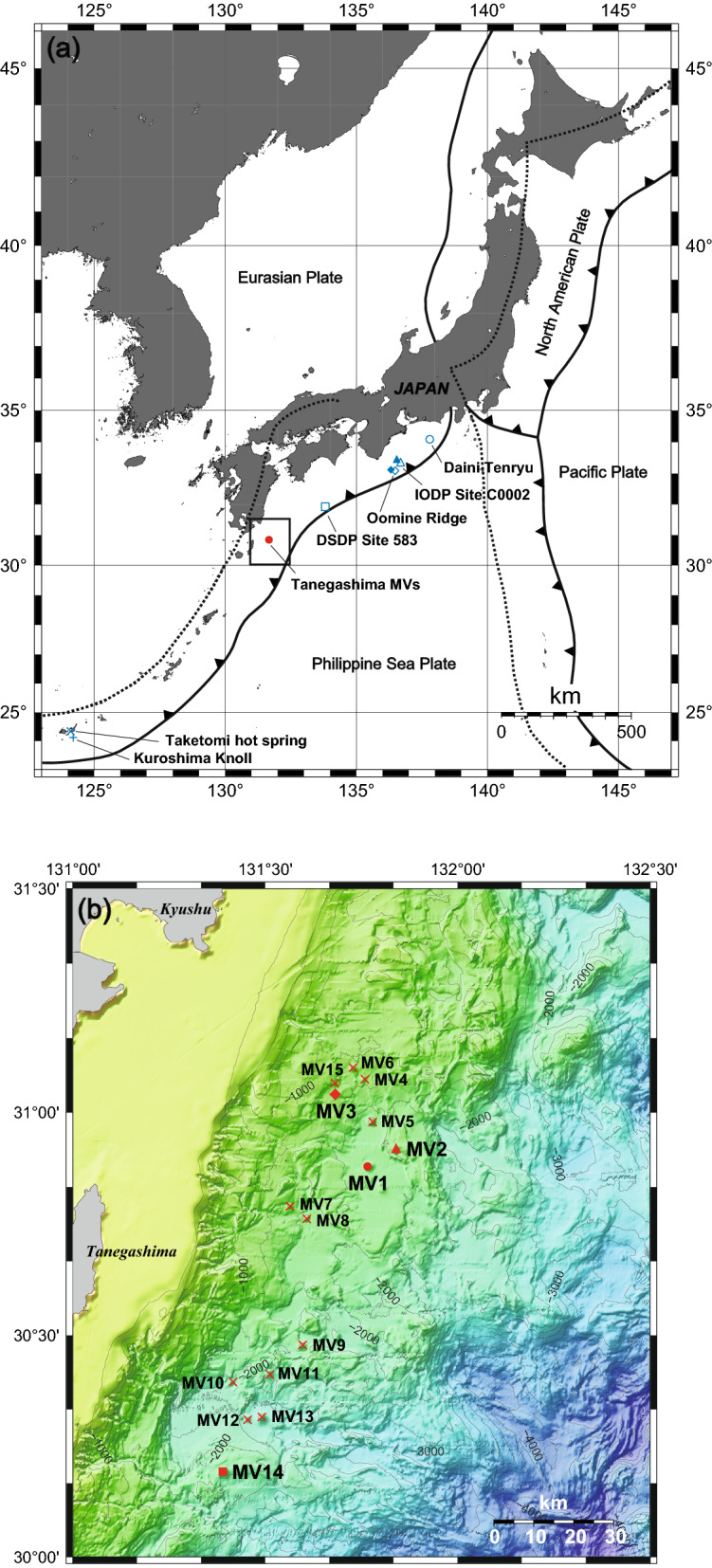
(**a**) Map showing the plate tectonics around Japan and the sampling sites in this study. The sampling points of the data used in the discussion are also shown as blue points for comparison (for the shape of the symbols, please refer to the legend in Fig. 3). The solid black line indicates the plate boundary, and the dotted black line indicates the volcanic front^31,32^. The squared area indicates the area shown in (**b**). (**b**) Seafloor topographic map of the area around the submarine mud volcano off Tanegashima Island studied in this study. The filled red circle indicates the location of MV1, the filled red triangle indicates MV2, the filled diamond indicates MV3, and the filled red square indicates MV14. Other submarine mud volcanoes are marked by red crosses. The map was drawn using Online Mapping Tools for GMT (https://www.generic-mapping-tools.org/).

Tanegashima Island is located in southwestern Japan (Fig. 1a) in a plate convergence zone where the Philippine Sea plate is subducting into the Eurasian plate at a rate of 4.6 cm per year^24^. This plate convergence zone is part of the erosional Ryukyu Trench^25^. According to the *P*-wave velocity models in the surrounding subseafloor crust, the plate boundary around Tanegashima Island is thought to be located approximately 18 km below the seafloor, and the upper part of the plate is classified into the upper, middle, and lower crust, as often occurs in the structure of island arcs^26^. The geological basement of the surrounding area consists of the Eocene Kumage Group, which is equivalent to the Shimanto Formation, and subduction-related thrust faults and folds have been observed on land^27^. Eocene foraminifera have been obtained from the surface layers of mud volcanoes, suggesting that the Kumage Formation is contemporaneous with the deeper layers of the mud volcanoes^22^. A side-scan sonar survey conducted in 1990 covered an area 20–30 km wide and 1200–3500 m deep from the northern end of the Ryukyu Trench slope to the southern end of the Ryukyu Trench slope revealing more than 30 mud diapirs in the northern part of the trench slope^22^. Bathymetric and submarine tectonic surveys conducted between 2012 and 2014 have thus far identified 15 submarine mud volcanoes, named MV1-MV15 in the order in which they were found (Fig. 1b)^28,29^. In MV2 and MV13, Quaternary hemipelagic sediments overlie Cretaceous and Neogene sediments, suggesting that mud volcanism may have been stagnant at the time of the 1994 survey^22^. The sedimentation rate of those Quaternary deposits was estimated to be less than 12–15 cm/ka in MV2 and approximately 15 cm/ka in MV13^22^. In the 2002 survey, the chemical and isotopic compositions of porewater in the surface sediments of MV1 showed a distribution of water of dehydration origin in clay minerals, suggesting that water was supplied from deep beneath the seafloor, at least as early as 2002 when the sampling was conducted^23^. In MV1 and MV14 during the survey in 2015, a part of the results of this study, high concentrations of methane and microflora characteristic of surface sediments were observed in the seawater column several tens of meters above the summit, suggesting that these mud volcanoes were active at least immediately before the 2015 sampling campaign (see below)^30^.

## Sampling and analytical method

Samples were collected on the RV Hakuho Maru (expedition KH-15-2) from August 19th to September 1st, 2015, and from August 9th to August 18th, 2019 (expedition KH-19-5) (Table 1). Due to ship time, only two sampling sites could be carried out on each voyage. Sampling was also limited to the tops of some mud volcanoes, e.g., MVs 1 and 2, because Japanese marine survey rules require a distance of twice the water depth from the submarine cable. For the sampling in 2015, small 55-cm-long sections of the material collected with the piston core at MV1 and MV14 were cut from each PVC liner containing sediment, and PVC pistons with O-rings were placed as seals to break contact with the atmosphere at both ends of the sediments^33^. Holes were drilled into the sides of the PVC liner, and copper tubes (diameter: 1 cm, length: 60 cm) were attached using stainless-steel fittings. A PVC piston attached to each end pushed the bulk sediment into the copper tubes. The copper tubes were flushed with sediments to minimize air contamination and then sealed by pinching both ends with metal clamps^34^. For the 2019 sampling, surface sediments from MV2 and MV3 were collected using a 60-cm-long multiple corer, which was processed to avoid more atmospheric contamination, and copper tubes were attached to predrilled side holes^35^. All sediment samples were further processed onshore. To this end, the copper tubes underwent centrifugation to separate the porewater from the sediment matrix. Subsequently, noble gas analysis was conducted on the porewater phase^34^. Samples collected in 2015 were analyzed in the Noble Gas Laboratory at the Swiss Federal Institute of Technology Zurich (ETHZ) using two custom-made magnetic sector mass spectrometers^36^. For Ar, ^36^Ar and ^40^Ar were measured and summed up as the Ar concentration; for Kr and Xe, ^86^Kr and ^136^Xe were measured and converted to Kr and Xe concentrations assuming the same composition as that of the atmosphere^37^. Samples collected in 2019 were analyzed at the Atmosphere and Ocean Research Institute (AORI) of the University of Tokyo by a noble gas mass spectrometer (Helix SFT; GV Instruments Ltd.; Manchester, UK) for He concentration and isotope ratios and a quadrupole mass spectrometer for ^20^Ne/^4^He ratios (Prisma 80; Pfeiffer Vacuum; Asslar, Germany)^38^. Atmospheric air was used as the standard gas to calibrate the measurements. The respective analytical errors are shown in Table 2. Sediment samples for porosity measurements were subsampled from cores collected from MV14. Samples were sealed in plastic bags and stored frozen, and wet and dry weights were measured. The porosity was approximated based on definitions and with a number of assumptions (Supplement S1)^39^.

**Table 1:** Sampling sites, dates, latitude, longitude, depth, and total length of cores collected from surface sediments of submarine mud volcanoes off Tanegashima Island in this study.

Site	Date	Latitude	Longitude	Depth	Core length
		N	E	m	cm
MV1	August 23, 2015	30°52.71′	131°46.01′	1415	338
MV2	August 16, 2019	30°55.1158′	131°50.3944′	1366	26
MV3	August 17, 2019	31°02.5373′	131°40.9536′	1136	27
MV14	August 30, 2015	30°11.46′	131°23.58′	1693	311

**Table 2:** Helium isotope ratios (^3^He/^4^He), neon/helium ratio (^20^Ne/^4^He), argon isotope ratio (^40^Ar/^36^Ar) and concentrations of argon, krypton and xenon in surface sediments of submarine mud volcanoes off Tanegashima Island obtained in this study.

Sample	Depth	Pore water	(^3^He/^4^He )_obs_	^20^Ne/^4^He	^40^Ar/^36^Ar	Ar	Kr	Xe
	cmbsf	g	R_A_		× 10^2^	× 10^–4^ cm^3^_STP_/g	× 10^–8^ cm^3^_STP_/g	× 10^–9^ cm^3^_STP_/g
MV1	300	0.89 ± 0.05	0.23 ± 0.05	0.230 ± 0.120	3.22 ± 0.05	0.96 ± 0.01	1.49 ± 0.03	2.02 ± 0.05
MV2	18	2.29 ± 0.03	0.63 ± 0.01	0.271 ± 0.027	N.A	N.A	N.A	N.A
MV3-1	8	2.27 ± 0.03	0.34 ± 0.01	0.054 ± 0.005	N.A	N.A	N.A	N.A
MV3-2	13	2.64 ± 0.03	0.42 ± 0.01	0.276 ± 0.028	N.A	N.A	N.A	N.A
MV3-3	18	3.17 ± 0.03	0.74 ± 0.02	1.24 ± 0.12	N.A	N.A	N.A	N.A
MV14	274	15.48 ± 0.05	0.95 ± 0.01	0.835 ± 0.002	3.04 ± 0.01	1.050 ± 0.001	2.62 ± 0.01	4.72 ± 0.06
ASW	–	–	1	4.0	2.96^a^	3.61^b^	8.84^c^	13.56^d^

Data for seawater in equilibrium with the atmosphere are also shown.

N.A. : Not analyzed.

^a^Ozima and Podosek^37^.

^b^Weiss^40^.

^c^Weiss and Kyser^41^.

^d^Jenkins et al.^42^.

## Results

The weight of porewater analyzed in this study (g), the He isotope ratio in porewater ((^3^He/^4^He)_obs_; R_A_), the ^20^Ne/^4^He ratio and the concentrations of Ar, Kr and Xe (cm^3^_STP_/g) are reported in Table 2. The noble gas concentrations of air-saturated seawater (ASW) shown in the table were calculated based on the parameterizations by Weiss^40^ for Ar, Weiss and Kyser^41^ for Kr and Jenkins et al.^42^ for Xe. The physical conditions used for the ASW calculations were the temperature (2 °C) and salinity (35 psu) near the seafloor, as observed during expedition KH-15–2 and an atmospheric pressure of 1 bar. The measured (^3^He/^4^He)_obs_ ranged from 0.23 ± 0.05 to 0.95 ± 0.01 R_A_, i.e., below the ASW value of 1 R_A_ (Table 2). MV1 had the lowest He isotope ratio of 0.23 ± 0.05 R_A_ and MV14 had the highest He isotope ratio of 0.95 ± 0.01 R_A_. At MV3 the measured He isotope ratios increased with increasing depth: 0.34 ± 0.01 R_A_ at 8 cmbsf, 0.42 ± 0.01 R_A_ at 13 cmbsf and 0.74 ± 0.02 R_A_ at 18 cmbsf. The (^3^He/^4^He)_obs_ ratio at MV2 was 0.63 ± 0.01 R_A_. The ^20^Ne/^4^He values showed that MV3-1 had the lowest value of 0.054 ± 0.005, followed by MV1, MV2, MV3-2, and MV14, with MV3-3 having the maximum value of 1.24 ± 0.12 (Table 2). In these mud volcanoes, the measured ^20^Ne/^4^He values were significantly lower than the ASW beyond the error ranges. Comparing the ^20^Ne/^4^He values of the three MV3 samples, MV3-1 < MV3-2 < MV3-3, the deeper layers had higher values. Two ^40^Ar/^36^Ar ratios could be determined, 3.22 ± 0.05 × 10^2^ (MV1) and 3.04 ± 0.01 × 10^2^ (MV14), which were slightly lower than the ratio for ASW (2.96 × 10^2^) (Table 2). The respective Ar concentrations, 0.96 ± 0.01 × 10^−4^ cm^3^_STP_/g (MV1) and 1.050 ± 0.001 × 10^−4^ cm^3^_STP_/g (MV14), were found to be lower than that expected in ASW (3.61 × 10^−4^ cm^3^_STP_/g; Table 2). The concentration of Kr was 1.49 ± 0.03 × 10^−8^ cm^3^_STP_/g for MV1 and 2.62 ± 0.01 × 10^−8^ cm^3^_STP_/g for MV14, i.e., both were lower than the Kr concentration of ASW (8.84 × 10^−8^ cm^3^_STP_/g; Table 2). The Xe concentrations of MV1 and MV14 were 2.02 ± 0.05 × 10^−9^ cm^3^_STP_/g and 4.72 ± 0.06 × 10^−9^ cm^3^_STP_/g, respectively, which were one order of magnitude lower than the Xe concentration of ASW (13.56 × 10^−9^ cm^3^_STP_/g; Table 2). The concentrations of Ar, Kr and Xe were higher in MV14 than in MV1 (Table 2).

## Discussion

### Effects of atmospheric origin components

**Figure 2:**
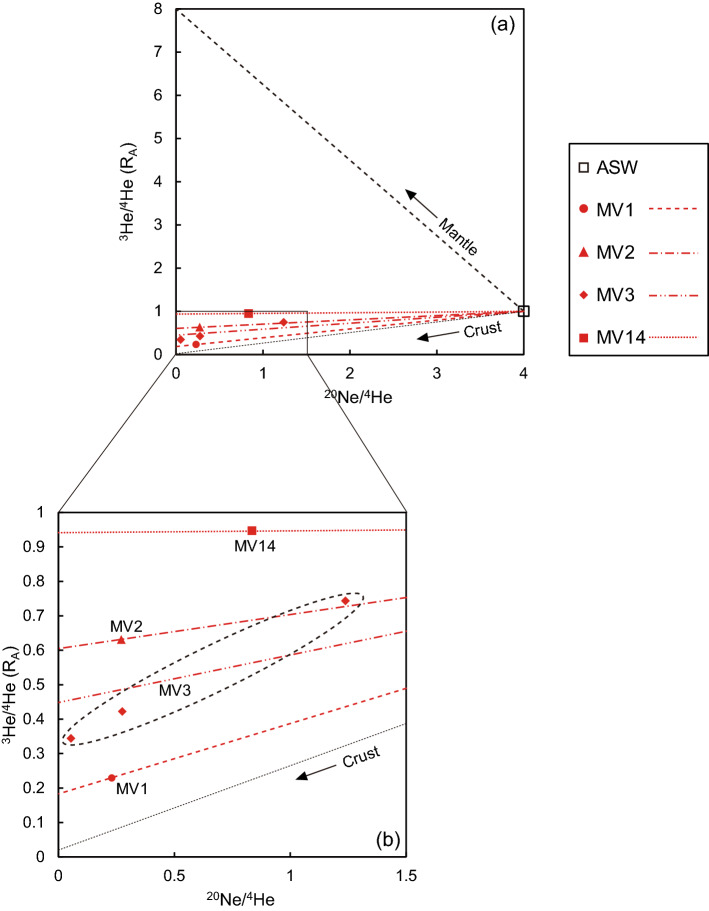
(**a**) Relationship between He isotope ratios (^3^He/^4^He) and Ne–He ratios (^20^Ne/^4^He) of gases in surface sediments of submarine mud volcanoes off Tanegashima Island. Black dashed and black dotted lines are mixing lines of mantle-derived He and air-saturated seawater (ASW) and of crust-derived He and ASW, respectively. The red dashed line shows the mixing line of ASW and MV1, the single-dotted line shows the mixing line of MV2, the double-dotted line shows the mixing line of MV3, and the dotted line shows the mixing line of MV14. (**b**) Expanded view of the plot area of the samples in (**a**). The MV3 data are surrounded by a black dotted ellipse because they vary slightly.

The ^20^Ne/^4^He ratio^43,44^ was used to evaluate the mixing ratio of fluids originating from the solid Earth and seawater that has reached equilibrium with the atmosphere (ASW). Here, ^20^Ne is overwhelmingly more abundant in the atmosphere than in the mantle and crust, and therefore ^20^Ne in the solid earth can be neglected. Figure 2 shows the relationship between ^3^He/^4^He and ^20^Ne/^4^He in the gases obtained in this study. Here, the noble gases of mantle origin are assumed to be characterized by an isotope signature close to (^3^He/^4^He, ^20^Ne/^4^He) = (8 R_A_, 0), those of crustal origin by (^3^He/^4^He, ^20^Ne/^4^He) = (0.02 R_A_, 0), and those of atmospheric origin by (^3^He/^4^He, ^20^Ne/^4^He) = (1 R_A_, 4). The values of the ^20^Ne/^4^He ratio of ASW were calculated by assuming the respective atmospheric abundances and the appropriate temperature and salinity. First, the seawater mixed into the porewater at the bottom of the deep sea is considered to be bottom seawater, and the concentration of noble gases in the seawater is almost uniform, with a variation of only a few percent^45^. The ^20^Ne/^4^He ratio calculated under these conditions was 3.60 to 3.69^37,46,47^. The temperature of the surface seawater in this area is considered to be about 20 to 25 °C^48^ and salinity 34 to 35 psu^49^. The ^20^Ne/^4^He ratio calculated under the temperature (2 °C) and salinity (35 psu) of the bottom water observed at the site in this study is 4.00^37,46,47^. Considering these facts, the ^20^Ne/^4^He ratio is considered to be 4 here. The red dashed, single-dashed, double-dashed, and dotted lines are straight lines connecting the noble gas data for mud volcanoes MV1, MV2, MV3, and MV14 with the ASW values, respectively (Fig. 2). For MV3, a regression line through the ASW is drawn for the three samples of data (Fig. 2). The tritium concentration in deep seawater is so low that the tritium-derived ^3^He contribution is almost negligible^50^. This result suggests that the He in the gas collected in this study can be explained by a mixture of crustal, mantle, and atmospheric equilibrium seawater sources (Fig. 2).

**Figure 3:**
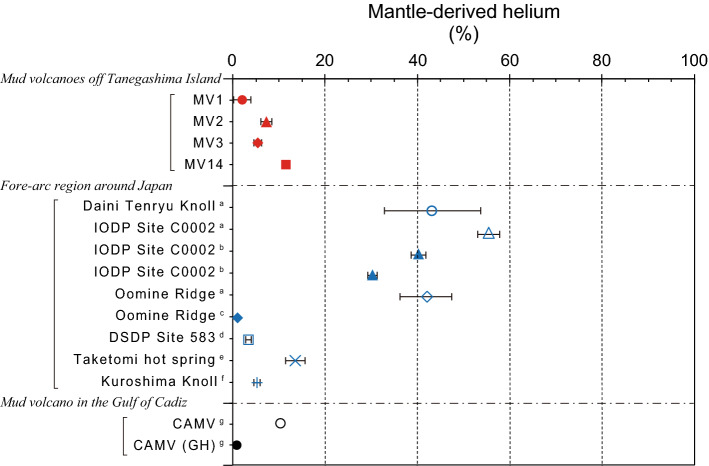
Fractions of mantle-derived He in the surface sediments of the submarine mud volcano off Tanegashima Island estimated in this study. Also plotted are the fractions of mantle-derived He in the sediments in the forearc region around Japan and the fraction of mantle-derived He in the surface sediments of submarine mud volcanoes in the Gulf of Cadiz. References are, respectively, as follows: a: Tomonaga et al.^50^, b: Wiersberg et al.^56^, c: Toki et al.^55^, d: Sano and Wakita^57^, e: Toki et al.^58^, f: Tsunogai et al.^59^, g: Nuzzo et al.^20^.

In Fig. 2, the horizontal axis can be thought of as the contribution of noble gases of atmospheric origin, such as the atmosphere or ASW: the closer a value is to the air or ASW value, the greater the influence of the atmospheric component (Fig. 2). We can estimate the atmosphere-derived share of noble gases in the pore fluids by considering binary mixing of a ^20^Ne-free component and an ASW component^43,44^. In the case of this study, since the samples were collected from the seafloor and we were very careful to avoid atmospheric contamination during sampling and analysis, we assume that all the ^20^Ne originated from ASW. Fraction A of the non-atmospheric and atmosphere-derived noble gases in the measured samples can be expressed as

1 $${\text{A}} = (^{20} {\text{Ne}}/^{4} {\text{He}})_{{{\text{obs}}}} /(^{20} {\text{Ne}}/^{4} {\text{He}})_{{{\text{asw}}}}$$

where (^20^Ne/^4^He)_obs_ is the observed ^20^Ne/^4^He ratio and (^20^Ne/^4^He)_asw_ is the value of ASW as described earlier ((^20^Ne/^4^He)_asw_ = 4). Table 3 summarizes the obtained values for A. According to this result, the values of A ranged from 0.014 ± 0.001 to 0.310 ± 0.031, indicating a range of 1.4 ± 0.1 to 31.0 ± 3.1% of gases with an atmospheric origin (Table 3). The largest A-value was observed for MV3-3 (0.310 ± 0.031) and the smallest A-value was for MV3-1 (0.014 ± 0.001) (Table 3).

**Table 3:** Atmospheric equilibrium seawater contribution estimated from ^20^Ne/^4^He in surface sediments from submarine mud volcanoes off Tanegashima Island (A) and helium isotopic ratios ((^3^He/^4^He)_corr_) corrected for the atmospheric equilibrium seawater contribution, and mantle-derived and crust-derived helium contribution estimated from the corrected values (M and C, respectively).

Sample	A	(^3^He/^4^He )_corr_	Mantle	Crust
		R_A_	%	%
MV1	0.058 ± 0.030	0.18 ± 0.17	2.0 ± 1.8	98.0 ± 1.8
MV2	0.068 ± 0.007	0.60 ± 0.10	7.3 ± 1.2	92.7 ± 1.2
MV3-1	0.014 ± 0.001	0.34 ± 0.05	4.0 ± 0.6	96.0 ± 0.6
MV3-2	0.069 ± 0.007	0.38 ± 0.06	4.5 ± 0.7	95.5 ± 0.7
MV3-3	0.310 ± 0.031	0.63 ± 0.10	7.6 ± 1.2	92.4 ± 1.2
MV14	0.209 ± 0.001	0.93 ± 0.02	11.5 ± 0.2	88.5 ± 0.2

In general, the seafloor is in contact with seawater at the seafloor surface, so seawater intrudes through the seafloor surface, and thus the influence of seawater would be greater in the surface layer. But MV3-1 has the least influence of seawater despite being in the surface layer, and the deeper MV3-3 has the greatest influence of seawater (Table 3). This may be because, unlike normal depositional processes, sediment disturbance occurs as a depositional process specific to the mud volcano surface layer. The small influence of seawater means that the influence of gas from the deep layer is relatively large, and it is thought that mud volcanism is the driving force of gas migration from the deep layer, suggesting that MV3 is the most active mud volcano. On the other hand, MV14, which was suggested to be the most intruded by seawater, is considered to be the most inactive mud volcano among the four mud volcanoes off Tanegashima sampled in this study. However, as observed in MV3, the vertical distribution of the noble gas isotope signature in the pore space should be highly heterogeneous. In addition, MV14 may also be heterogeneous within a single mountain body with respect to the horizontal direction, considering that methane and microbial plumes were observed in seawater a few 10 m below the seafloor near the summit^30^. For example, data with large anomalies may be obtained near the crater, but if the samples are collected at a distance from the crater, it is possible that the activity level may be underestimated. In light of these considerations, assessing the activity level of a mud volcano based on a very limited number of samples may be an error in evaluation and should be done with caution. More noble gas data needs to be acquired in the future.

### Origin of He

As described above, the contribution of atmospheric He among the three sources of noble gases was quantitatively evaluated using the ^20^Ne/^4^He ratios (Table 3). The other sources are all related to the source of He in the Earth's interior and consist of He from deeper mantle sources and He of crustal origin that are mixed in during the ascent of fluids through the crust. Hence the He isotope ratios can be corrected as follows (Eq. 2)^51^.

2 $$(^{3} {\text{He}}/^{4} {\text{He}})_{{{\text{corr}}}} = ((^{3} {\text{He}}/^{4} {\text{He}})_{{{\text{obs}}}} - (^{3} {\text{He}}/^{4} {\text{He}})_{{{\text{asw}}}} \times {\text{A}})/(1 - {\text{A}})$$

The (^3^He/^4^He)_asw_ represents the He isotope ratio of ASW (= 1.39 × 10^–6^: 1 R_A_). On the other hand, (^3^He/^4^He)_obs_ represents the observed He isotope ratio, and (^3^He/^4^He)_corr_ is the corrected He isotope ratio after subtracting the atmosphere-derived He share. The calculation process is shown in Supplement S2, and the results are shown in Table 3.

The (^3^He/^4^He)_corr_ thus obtained can be expressed by a mixture of mantle and crustal He as follows:

3 $$(^{3} {\text{He}}/^{4} {\text{He}})_{{{\text{corr}}}} = (^{3} {\text{He}}/^{4} {\text{He}})_{{\text{m}}} \times {\text{M}} + (^{3} {\text{He}}/^{4} {\text{He}})_{{\text{c}}} \times {\text{C}}$$

4 $${\text{M}} + {\text{C}} = 1$$

where (^3^He/^4^He)_m_ and (^3^He/^4^He)_c_ are the He isotope ratios of mantle-derived He and crustal He, respectively, using 8 R_A_ and 0.02 R_A_, respectively^52–54^. M and C are the fractions of mantle-derived He and crustal-derived He, respectively. The inferred (^3^He/^4^He)_corr_ values are summarized in Table 3. These values correspond to the y-intercepts of the linear regressions in Fig. 2. (^3^He/^4^He)_corr_ values range between 0.18 ± 0.17 and 0.93 ± 0.02 R_A_, with MV1 being characterized by the lowest value (0.18 ± 0.17 R_A_), followed by MV3 with values between 0.34 ± 0.05 and 0.63 ± 0.10 R_A_, followed by MV2 with 0.60 ± 0.10 R_A_, and finally MV14 had the largest value (0.93 ± 0.02 R_A_). The contribution of mantle-derived He ranges from 2.0 ± 1.8 to 11.5 ± 0.2%, with the lowest contribution at MV1 and the highest contribution at MV14 (Table 3). Thus, nearly 90% of He is crustal in origin (Table 3). This indicates that the main source of crustal-derived He is located in the crust rather than in the mantle. The contribution of mantle-derived He at MV14 was slightly higher than that of the other mud volcanoes in this study, suggesting that deeply seated fluids may be supplied to the surface sediments of MV14 to a slightly greater extent than those of the other mud volcanoes in this study (Table 3).

The R/R_A_ and thus the contribution of mantle derived He were compared with reported values of He isotope ratios, such as those in cold seeps and borehole fluids in the forearc region of Japan (Fig. 3; Supplement S2)^20,50,55–59^. We used the average value for MV3 for the submarine mud volcanoes off Tanegashima Island obtained in this study, since there are three datasets at three different depths. In addition, we also included data from the Gulf of Cadiz (CAMV), where noble gases were studied with respect to submarine mud volcanoes^20^. In the Gulf of Cadiz, the ^4^He concentration in the gas associated with gas hydrates is observed and treated separately, so it is plotted separately as the CAMV (GH) in this study (Fig. 3).

These forearc regions generally have limited mantle He input^7^. However, there are some cases reported where special faults act as supply channels for mantle-derived He^7^, and in such locations the contribution of mantle-derived He exceeds 30% (Fig. 3). On the other hand, in mud volcanoes such as the present study and CAMV, the contribution is ~ 10% at most, which is not particularly high (Fig. 3; Supplement S2). The contribution of mantle-derived He to the He supplied to the surface sediments of mud volcanoes may be less than 20% (Fig. 3). However, since mud volcanoes are active and inactive over rather variable periods of time, it is too early to determine the general characteristics of mud volcanoes from these data alone, and hence, more empirical evidence is needed.

### Depth of origin estimated from the dissolution equilibrium of heavy noble gases

The distribution of heavy noble gases (Ar, Kr, and Xe) in the ocean can be explained by supersaturation in the surface layer due to gas exchange with the atmosphere and bubbles, and under saturation in the deeper layers due to rapid cooling in the general ocean circulation^45^. However, this supersaturation and unsaturation is only a few percent of the atmospheric equilibrium concentration, and it is safe to assume that in ocean water, from the surface to the deep layer, the atmospheric equilibrium concentration is almost constant within the margin of error. In considering porewater, only ^36^Ar, which is not produced in the crust, will be considered in this study, since ^40^Ar is produced in the crust therefore increasing its concentration in the crust^37^. The heavier noble gases in the porewater are dissolved gases that are originally found in deep seawater and buried at a rate of approximately 0.1 mm per year in this region^22^. However, in the sediments, ambient temperature increases slightly with burial depth due to a geothermal gradient (25 °C/km in the study area)^60^. As the temperature increases, the gas solubility decreases^40^. After the deep seawater is buried, the gas is distributed according to the temperature conditions at different depths. Therefore, we calculated the dissolution equilibrium temperature corresponding to the concentration of heavy noble gases observed in the field.

The dissolution equilibrium temperatures in each element are based on ^36^Ar^37,40^, Kr^41^ and Xe^42^. First, the ^36^Ar concentration was calculated from the obtained ^40^Ar/^36^Ar ratio and Ar concentration (Table 2). Next, the amount of ASW contamination was corrected using ASW fraction A (Table 3) estimated from the ^20^Ne/^4^He ratio, as follows:

5 $${\text{X}}_{{{\text{corr}}}} = ({\text{X}}_{{{\text{obs}}}} - {\text{X}}_{{{\text{asw}}}} \times {\text{A}})/(1 - {\text{A}})$$

**Figure 4:**
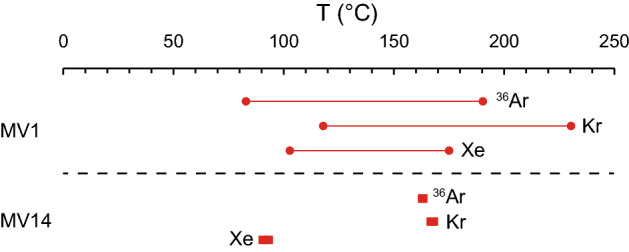
Dissolution equilibrium temperatures estimated from observed noble gas concentrations at MV1 and MV14.

In this case, X represents any noble gas species, the subscript corr represents the corrected value, obs represents the observed value, and asw represents the atmospheric equilibrium seawater. The corrected values (^36^Ar_corr_, Kr_corr_ and Xe_corr_) are summarized in Supplement S3. Next, note that salinity is required for solubility equilibrium concentration calculations. The in situ salinity during gas exchange at depth was assumed to be pure water (0 psu) as coming from a fluid of clay mineral dehydration origin based on the previous study^23^. The dissolution equilibrium temperatures obtained under these assumptions show different temperature ranges for each element (Supplement S3; Fig. 4), ranging from 83 to 190 °C (MV1) and 162.8 to 163.4 °C (MV14) for ^36^Ar, 118 to 230 °C (MV1) and 166.7 to 168.1 °C (MV14) for Kr, and 103 to 175 °C (MV1) and 90.6 to 93.3 °C (MV14) for Xe. The calculation process is shown in Supplement S4. Note that seawater does not reach its boiling point under these temperature and pressure conditions^61^. It should be noted that such a rapid decrease in concentration may be observed even in the case of inflow of porewater in which a free gas phase has formed and the gas concentration has decreased. For example, in areas where natural gas is abundant, such a free gas phase may form in the porewater, resulting in secondary gas distribution of noble gases. However, the results from seismic surveys beneath the seafloor do not suggest the presence of free gas^28,29^. Here, the presence of free gas is not considered. It should be noted that empirical gas solubility data have been obtained only for temperature conditions of up to approximately 30 °C^40–42^. Therefore, their application to higher temperature ranges is subject to a rather large uncertainty. In the future, solubility in seawater under high temperature and high-pressure conditions should be supported through laboratory experiments and simulations. For example, it has been shown that the solubility of noble gases is slightly higher in the presence of supercritical carbon dioxide^62^. Taking this into account, the temperature estimates made in this study could be revised as slightly higher.

For MV1 the temperature range could be better constrained. This is a problem of error propagation in the estimation, especially in this case because the error in the measured ^20^Ne/^4^He ratio was much larger for MV1 (Table 2), which caused the error in the corrected ASW contamination value using the ASW fraction to be larger. For MV14, the temperatures estimated for each element did not agree with each other, but for MV1, 118 to 175 °C could be the temperature equilibrium for any element (Supplement S3). The temperature ranges estimated for MV1 (83 to 230 °C) and MV14 (91 to 168 °C) reflect the temperature range at which dehydration reactions can occur in clay minerals (60 to 160 °C)^63^ and were calculated using the previously reported geothermal gradient in this area (25 °C/km)^60^. The subseafloor depths that could have these temperature ranges were 3.3 to 9.2 km (MV1) and 3.6 to 6.7 km (MV14), respectively (Supplement S3). The origin depth estimated in this study corresponds to the acoustic intermediate crust, since the plate boundary is estimated to be 18 km below the seafloor based on the sound velocity structure of the surrounding crust^26^. This is consistent with the fact that nearly 90% of the He originates from the crust (Table 3).

## Implications for mud volcanic activity indicated by noble gas data

In this study, sediments were collected from the surface layer of submarine mud volcanoes off Tanegashima Island, and the concentrations and isotopic ratios of noble gases in the sediments were determined. Within this paper, the following findings have been made thus far.

Based on the ^20^Ne/^4^He values, the surface layer of MV14 is stagnant in mud volcanic activity due to significant seawater intrusion (Section “Effects of atmospheric origin components”).

Based on ^3^He/^4^He values, the contribution of He from the mantle to the surface layer of MV14 is the largest in the present study (Section “Origin of He”).

**Figure 5:**
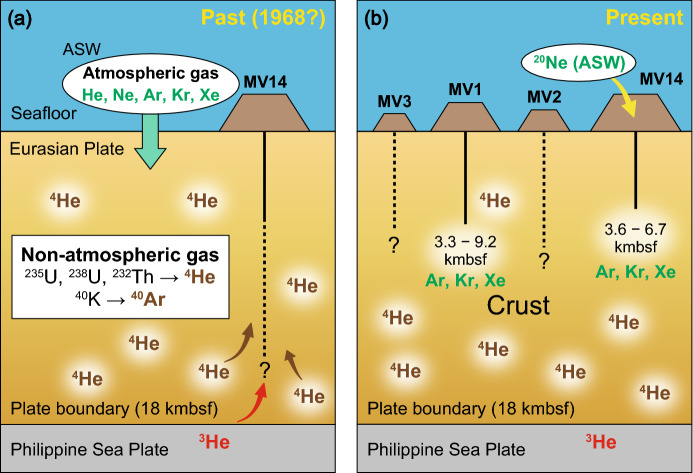
Depth of the origin of noble gases (**a**: past, **b**: present) estimated from the distribution of noble gases in surface sediments collected from submarine mud volcanoes off Tanegashima Island studied in this study.

The largest contribution of mantle-derived He suggests that the gas was supplied from the deepest depths with significant energy. This is seemingly contradictory given the stagnation of MV14 activity. However, this stagnation may be due to a time lag between the main period of activity and the transport of gases within the sediment column during stagnation (Fig. 5). Thus, the higher He isotope ratio at MV14 could represent a remnant of past eruptions.

First, MV14 is the largest mud volcano among those included in this study (Supplement S5). It is possible that MV14 was once active to the point of becoming a large body, but is already a dormant mud volcano. During its active phase, there may have been a supply of mantle-derived He from deep within the crust. The observed facts can be explained by the scenario that the activity of MV14 has since decayed, as indicated by the Ne concentration, to the point where seawater is now intruding, but that traces of the former mantle-derived He still remain.

Assuming that such an eventful noble gas was supplied for a period of time, we estimated how many years He would remain undiffused until it was detected as a trace at the time of sampling (Eqs. 6–8)^64,65^.

6 $$\Delta z_{diff} \approx \left( {\varphi D\Delta t} \right)^{1/2}$$

7 $$D = D^{0} /a$$

8 $$D^{0} = Ae^{ - Ea/RT}$$

where Δ*z*_*diff*_ is the diffusion distance (cm), *φ* is the porosity in the sediment, *D* is the effective diffusivity (cm^2^/s), Δ*t* is the time elapsed from the last supply of mantle-derived He until sample collection (sec), *D*^0^ is the molecular diffusion coefficient, *a* is an attenuation factor, *A* is the diffusion constant (cm^2^/s), *E*_*a*_ is the diffusion activation energy (J/mol), *R* is the gas constant (J/mol/K), and *T* is temperature (in K). The values used in the calculations are as follows: Δ*z*_*diff*_ is the depth at which samples were collected at MV14, at 274 cm; *φ* is the mean porosity of 40.4 ± 3.9% of the surface sediments collected at MV14 (see Supplement S2); *a* is 2^64,66,67^,* A* is the diffusion constant of He (8.86 × 10^–3^ cm^2^/s)^65^ and diffusion activation energy (12.02 kJ/mol)^65^. The water temperature (2 °C) and a geothermal gradient of 25 °C/km^60^ were used to calculate a temperature at 274 cmbsf (2.1 °C). *D*^0^ is the diffusion coefficient of He, and was calculated to be 4.64 × 10^–5^ cm^2^/s (Eq. 8). *D* was calculated to be 2.32 × 10^–5^ cm^2^/s (Eq. 7). The Δ*t* obtained using these values is 8.01 ± 0.80 × 10^9^ s (Eq. 6), corresponding to 254 ± 25 years. Here, the error of Δ*z*_*diff*_ accounted for the error in the depth direction of the sediment in the copper tube. If the volume of the copper tube (47.1 cm^3^) were to be concentric around the inlet of the copper tube, it would be approximately 2.8 cm hemispherical, so the error of the Δ*z*_*diff*_ of the sample was assumed to be ± 3 cm.

This estimated Δ*t* indicates that even if He is supplied from the mantle at 274 cmbsf, causing a concentration anomaly, no concentration anomaly at that depth will be detected after 254 ± 25 years. This suggests that the remaining mantle-derived He at 274 cmbsf must have been supplied within 254 ± 25 years. Sano et al.^68^ found traces of mantle-derived He in seawater immediately above the epicenter of the Great East Japan Earthquake, which was centered on the plate boundary off the Sanriku coast. They pointed out the existence of mantle-derived He that moved across the plate boundary at once^68^. Although not necessarily related to earthquakes, if a relatively large earthquake off Tanegashima Island triggered the supply of mantle-derived helium, for example, the most recent record of a relatively large earthquake off Tanegashima Island is the Hyuga-nada earthquake of magnitude 7.5 in 1968^69^. Prior to that, a large earthquake of magnitude 7.6 was reported to have occurred in 1662 in the same region^69–71^. Of these earthquakes, only the one in 1968 is considered to have occurred within a period of 254 ± 25 years. Other earthquakes in the M7.0–7.2 class occur about once every 20–27 years^72^, so one of these earthquakes may have triggered the supply of mantle-derived helium to the mud volcanoes. Monitoring mud volcanism will be necessary to clarify the relationship of such earthquakes with geochemical changes in the future. It has been noted that mantle-derived He moved across the plate boundary immediately after the earthquake^68^, and it will be necessary to further investigate the distribution of noble gases in mud volcanoes, including the time series.

## Conclusions

In this study, He isotope ratios and Ar, Kr, and Xe concentrations were measured in the porewater of sediments of submarine mud volcanoes off Tanegashima Island. The He isotope ratios in surface sediments of submarine mud volcanoes off Tanegashima Island suggest that approximately 90% of the He is of crustal origin, although there are differences in MV1 to MV3 and MV14. MV14 has the highest He isotope ratio among the four investigated mud volcanoes, suggesting that it may have once led to a plate boundary or fault, which could have acted as a preferential pathway for the transport of mantle-derived materials. Furthermore, the contribution of atmospheric equilibrium seawater suggests that MV14 is the least active mud volcano at present. The obtained concentrations of heavy noble gases in the pore water were used to estimate the dissolution equilibrium temperature with the fresh water source, which is consistent with the range of temperature conditions required for the dehydration of clay minerals. Converting this temperature range to the depth of origin based on the geothermal gradient reported for the area, it was estimated that dissolution equilibrium had been reached immediately prior at depths corresponding to 3.3–9.2 kmbsf in MV1 and 3.6–6.7 kmbsf in MV14. The origin of the heavy noble gases within the crust is consistent with the findings from the helium isotope ratios, as the plate boundary is estimated to be 18 kmbsf. The presented noble-gas dataset is rather limited but insights provided on fluid dynamics and geochemistry in mud volcanoes strongly motivate future research in this field.

## Supplementary Information

Supplementary Information 1.

Supplementary Information 2.

Supplementary Information 3.

Supplementary Information 4.

Supplementary Information 5.

## Data Availability

All data generated or analyzed during this study are included in this published article and its Supplementary Information files.
