# Supplementary material for: Estimation of the depth of origin of fluids using noble gases in the surface sediments of submarine mud volcanoes off Tanegashima Island
Source: Sci Rep. 2023 Apr 6;13:5051. doi: 10.1038/s41598-023-31582-z (PMC10079976; doi:10.1038/s41598-023-31582-z)
Supplement: Supplementary file 1 — Supplementary Information 1. [file 41598_2023_31582_MOESM1_ESM.docx]

**Estimation of the depth of origin using noble gases in the surface sediments of submarine mud volcanoes off Tanegashima Island**

Yuki Mitsutome, Tomohiro Toki, Takanori Kagoshima, Yuji Sano, Yama Tomonaga, Akira Ijiri

**Supplemental material**

S1: Porosity calculation

The weight of the wet sediment (Wet; g) in the sample bottle (Bottle; g) and the weight of the dry sediment (Dry; g) after drying were measured. Here, the weight of the wet sediment (Wet sediment; g) and the weight of the dry sediment (Dry sediment; g) can be obtained as follows.

Wet sediment (g) = Wet (g) - Bottle (g) (eq. S1)

Dry sediment (g) = Dry (g) - Bottle (g) (eq. S2)

Here, the difference in weight between the wet and dry sediment corresponds to the weight of water contained in the sediment (Water; g) and the volume of water (Water; cm^3^) can be determined using the density of water (σ_w_; g/cm^3^).

Water (cm^3^) = Water (g) / σ_water_ (g/cm^3^) (eq. S3)

Here, the density of the water is assumed to be 1.0 g/cm^3^.

The weight of salt (Salt; g) is then determined assuming that all the water is derived from the seawater that filled the pore spaces. Note that seawater is assumed to have a salinity of 35 psu.

Salt (g) / (Salt (g) + Water (g)) = 35 psu (eq. S4)

The volume of salt (Salt; cm^3^) was calculated using the density of salt (σ_salt_; g/cm^3^). The density of salt is 2.22 g/cm^3^.

Salt (cm^3^) = Salt (g) / σ_salt_ (g/cm^3^) (eq. S5)

The weight of the solid (Solid; g) is then obtained by subtracting the weight of salt (Salt; g) from the weight of the dry sediment (Dry; g)

Solid (g) = Dry (g) - Salt (g) (eq. S6)

Furthermore, the volume of solid (Solid; cm^3^) was calculated using the density of solid (σ_solid_; g/cm^3^). Here, the density of the solid is assumed to be 2.7 g/cm^3^.

Solid (cm^3^) = Solid (g) / σ_solid_ (g/cm^3^) (eq. S7)

Finally, the porosity (Φ) was calculated as the sum of the salt and water volumes (cm^3^) relative to the total volume (wet sediment; cm^3^) as follows.

Φ = (Salt (cm^3^) + Water (cm^3^)) / Wet sediment (cm^3^) (eq. S8)

The results are as follows (Table S1-1). The list of parameters used is also summarized in Table S1-2. These calculation methods follow the methodology used in the Integrated Ocean Drilling Program (IODP) (see Supplemental reference).

**Supplemental reference**

Expedition 320/321 Scientists. in *Proceedings of the Integrated Ocean Drilling Program* Vol. 332/321 (eds H. Pälike *et al.*) 80 (Integrated Ocean Drilling Program Management International, Inc., 2010).

Table S1-1: List of porosity values obtained. The mean and standard deviation of the obtained porosities are also given.

|  |  |  |  |  |  |  |  |  |  |  |  |  |  |  |
| --- | --- | --- | --- | --- | --- | --- | --- | --- | --- | --- | --- | --- | --- | --- |
| Sample ID | Wet | Dry | Bottle | Wet sediment | Dry sediment | Water | Water | Salt | Salt | Solid | Solid | Poro-sity | Porosity Average | Porosity Stdev. |
| Unit | g | g | g | g | g | g | cm^3^ | g | cm^3^ | g | cm^3^ |  |  |  |
|  |  |  |  |  |  |  |  |  |  |  |  |  |  |  |
|  |  |  |  |  |  |  |  |  |  |  |  |  |  |  |
| VTF5 4-1 | 11.9 | 11.0 | 8.75 | 3.10 | 2.30 | 0.807 | 0.807 | 0.029 | 0.013 | 2.27 | 0.84 | 49.4% | 40.44% | 3.94% |
| VTF5 4-2 | 12.4 | 11.7 | 8.75 | 3.63 | 2.95 | 0.679 | 0.679 | 0.025 | 0.011 | 2.92 | 1.08 | 38.9% |  |  |
| VTF5 4-3 | 11.5 | 11.0 | 8.75 | 2.74 | 2.29 | 0.455 | 0.455 | 0.016 | 0.007 | 2.27 | 0.84 | 35.5% |  |  |
| VTF5 4-4 | 11.6 | 11.0 | 8.75 | 2.82 | 2.28 | 0.545 | 0.545 | 0.020 | 0.009 | 2.26 | 0.84 | 39.8% |  |  |
| VTF5 5-1 | 11.2 | 10.7 | 8.75 | 2.47 | 1.93 | 0.537 | 0.537 | 0.019 | 0.009 | 1.91 | 0.71 | 43.5% |  |  |
| VTF5 5-2 | 11.5 | 10.9 | 8.75 | 2.73 | 2.14 | 0.588 | 0.588 | 0.021 | 0.010 | 2.12 | 0.78 | 43.2% |  |  |
| VTF5 5-3 | 11.8 | 11.2 | 8.75 | 3.07 | 2.46 | 0.600 | 0.600 | 0.022 | 0.010 | 2.44 | 0.90 | 40.3% |  |  |
| VTF5 5-4 | 11.8 | 11.3 | 8.75 | 3.04 | 2.50 | 0.534 | 0.534 | 0.019 | 0.009 | 2.48 | 0.92 | 37.1% |  |  |
| VTF5 6-1 | 12.1 | 11.5 | 8.75 | 3.35 | 2.75 | 0.601 | 0.601 | 0.022 | 0.010 | 2.72 | 1.01 | 37.7% |  |  |
| VTF5 6-2 | 11.8 | 11.3 | 8.75 | 3.08 | 2.53 | 0.546 | 0.546 | 0.020 | 0.009 | 2.51 | 0.93 | 37.4% |  |  |
| VTF5 6-4 | 11.9 | 11.2 | 8.75 | 3.10 | 2.46 | 0.643 | 0.643 | 0.023 | 0.011 | 2.44 | 0.90 | 42.0% |  |  |
|  |  |  |  |  |  |  |  |  |  |  |  |  |  |  |

Table S1-2: List of parameters used.

|  |  |  |
| --- | --- | --- |
| Parameter | Unit | Value |
|  |  |  |
|  |  |  |
| Salinity | g/g | 0.035 |
| Salt density | g/cm^3^ | 2.22 |
| Solid density | g/cm^3^ | 2.7 |
| Water density | g/cm^3^ | 1.0 |
|  |  |  |
